# Effect of the Japanese medical office system on job satisfaction, loyalty, engagement, and organizational commitment of medical practitioners: a survey of cardiologists in the acute care setting

**DOI:** 10.1186/s12913-023-10507-6

**Published:** 2024-01-26

**Authors:** Satoru Hashimoto, Yoshihiro Motozawa, Toshiki Mano

**Affiliations:** 1 Department of Healthcare Management, TCROSS Co., Ltd., NEOX Shinjuku Bldg. 7F, Shinjuku 1-9-1, Tokyo, 160-0022 Japan; 2 Graduate School of Strategic Management, Chuo University, Tokyo, Japan; 3 Department of Internal Medicine, San-ikukai Hospital, Tokyo, Japan

**Keywords:** Hospital reforms, Medical office system, Work engagement, Job satisfaction, Loyalty, Organizational commitment

## Abstract

**Background:**

In Japan, medical doctors have traditionally been assigned from university medical offices, under the medical office system. The present study examined the effects of the medical office system on job satisfaction, engagement, loyalty, and organizational commitment among cardiologists.

**Methods:**

In this study, a survey of 156 cardiologists was conducted, from April 22, 2023, to May 21, 2023, to examine the effect of the medical office system on employee job satisfaction, employee engagement, and organizational commitment.

**Results:**

Compared with the group that belonged to a medical office system (affiliated group, *n* = 117), the group that did not belong to a medical office system (non-affiliated group, *n* = 39) was affiliated to hospitals with a smaller number of beds. The results of the factor analysis showed that four types of hospital management styles were generated, namely, environment-, loyalty-building-, treatment-, and philosophy-oriented hospitals. There is an indication that the philosophy-oriented management style was adopted at the workplaces of the non-affiliated group. The treatment-oriented style also tended to be higher in the non-affiliated group than in the affiliated group. Furthermore, the non-affiliated group had higher organizational commitment, indicating that they were more likely to agree with the management philosophy set forth by hospital executives.

**Conclusion:**

Although the medical office system did not affect job satisfaction, compared with medical doctors with the affiliated group, those with the non-affiliated group tended to work in hospitals that emphasized philosophy-oriented management, and they received moderate compensation while practicing in an environment suitable for their specialty. These results suggest that the medical office system makes it difficult for medical doctors to have high workplace loyalty, engagement, and commitment to the hospital to which they are dispatched.

## Background

In Japan, medical doctors have traditionally been dispatched from university medical departments under the medical office system that is known for its highly bureaucratic style. Under the medical office system, a professor belonging to the same department in a university medical school is at the top of the organization, and is responsible for the personnel affairs of the medical doctors belonging to the medical office system [1]. Since 2004, the newly introduced post-graduate clinical training system for medical doctors has allowed residents to choose their training institutions. Although the medical office system appears to have weakened, several medical doctors still believe that there are advantages of being affiliated to the system after graduation. According to the Ministry of Health, Labour, and Welfare’s “Summary of the Results of the 2013 Clinical Training Completion Survey,” 4,047 (72.3%) of the 5,597 residents who received clinical training in the same year answered that they would “affiliate to the medical office system.” Moreover, of the 2,496 residents who completed their clinical training at university hospitals, 2,201 (88.2%) would “affiliate to the medical office.” In addition, 1,845 (59.5%) of the 3,101 doctors who completed their clinical training at non-university hospitals affiliated to the medical office system [2].

Since most medical doctors do not voluntarily decide where they will work but are assigned under the medical office system, their loyalty and work engagement with the hospital where they work may be low. Furthermore, in many cases, medical doctors are treated different from their co-medical and administrative staff in the hospital. In a survey conducted by a private research firm between April and May 2019, which received responses from 1,580 respondents, 46% said that they belonged to a university medical office system. Despite the wide age range of the medical doctors who responded to the survey, ranging in age from 30 to 60s, and the tendency for medical doctors to leave their medical offices and remain at their institutions as they get older [3], the effects of the medical office system on job satisfaction, engagement, and commitment are significant.

In this study, we examined the effects of the medical office system on job satisfaction, engagement, loyalty, and organizational commitment among cardiologists, who are leaders in acute care medicine, and identify the factors behind them.

## Methods

### Definitions

#### Medical office system

**Fig. 1:**
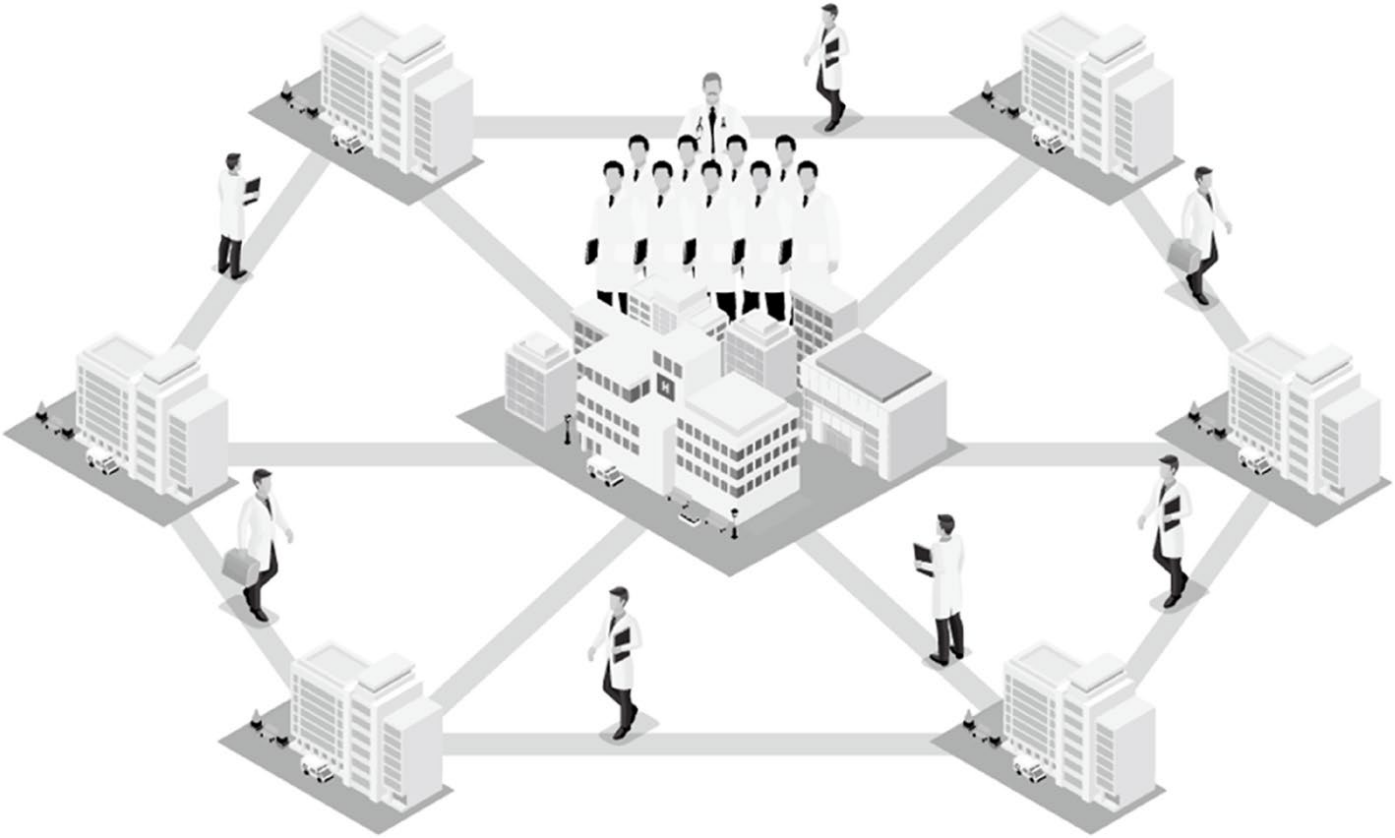
The diagram shows the medical office system in Japan, in which university hospitals take the lead in dispatching medical practitioners to affiliated hospitals. Doctors dispatched to affiliated hospitals move back and forth between the university hospital and the affiliated hospital when they are young while they gain experience as medical doctors

The medical office system is defined as “a system in which each department of a university establishes a close relationship with an affiliated hospital, thereby ensuring that the hospital has medical doctors, and that the university has high-quality hospitals [4].” In Japan, this system has long established the relationship between universities and affiliated hospitals. The top professors of the university departments hold authority over personnel affairs and support local medical services by dispatching medical doctors to affiliated hospitals (Fig. 1). This system can be described as a synonym for “membership-based employment” in which employment is secured by belonging to a pyramidal, seniority-based organization called the university medical office system. However, medical doctors who fall outside this system can be equated with “job-based employment,” where they can obtain result-based remuneration and positions by demonstrating their competence.

#### Employee engagement

Employee engagement is “a positive and fulfilling psychological state associated with work. characterized by vitality, enthusiasm, and immersion. Engagement is not a temporary state directed toward a specific object, event, individual, or behavior, but a sustained general feeling and perception directed toward work [5].”

#### Employee loyalty

Employee loyalty is the perception that employees are engaged in the success of the organization and that working for that organization is the best choice. The connection between the employee and organization is influenced by how the employee identifies with the combination of culture, structure, and leadership within the organization [6].

#### Organizational commitment

Organizational commitment is the sense of belonging and emotional attachment that employees feel toward the organization in which they work.

### Study population

This study investigated the relationship between employee engagement, loyalty, and organizational commitment with the medical office system through a web-based survey conducted using TCROSS NEWS, which is a news site specializing in cardiovascular medicine that began distribution in January 2010, with 90% of its medical doctor members being cardiologists. As of June 2020, 15,328 cardiologists belong to the Japanese Society of Cardiology, the largest medical academic organization in Japan [7], and as of July 31, 2022, the Japanese Association of Cardiovascular Intervention and Therapeutics has 8,448 regular members [8]. Therefore, 47.6% and 86.4% of the members of these 2 major societies, respectively, are considered registered on the site. The website’s membership is composed of the following: 3% of those in their 20s, 33% in their 30s, 33% in their 40s, 19% in their 50s, and 9% in their 60s. Moreover, approximately 70% of the members in their 30 s and 40 s are active on the front lines. In this study, of the approximately 8,000 medical doctor members of TCROSS NEWS, 3,500 individuals who wished to receive the TCROSS NEWS e-newsletter, from April 22, 2023, to May 21, 2023, were asked to complete questionnaires in a survey on medical doctor about employee engagement, loyalty to the hospital where they work, and employee satisfaction.

### Details of the survey

The survey included the following attributes: (1) present hospital, (2) number of beds at the hospital, (3) age of the participant, (4) sex of the participant, (5) presence and number of children, (6) affiliation with a medical office system, (7) length of service, (8) position at the hospital, (9) number of subordinates, and (10) job satisfaction on a five-point Likert scale. In addition, to investigate workplace conditions that include job satisfaction, employee engagement, and organizational commitment, questions related to (1) management philosophy (3 items), (2) organizational commitment (3 items), (3) external factors (5 items), (4) self-improvement (4 items), (5) working environment (3 items), and (6) environment for innovation creation (4 items) were independently generated based on information collected from previous studies and various sources [9–16].

### Statistical analysis

Descriptive statistics were calculated for each survey item. Categorical variables were compared using the chi-squared test or Fisher’s exact test, as appropriate. The Student’s t-test was used to compare continuous variables. A factor analysis with a maximum likelihood promax rotation was performed to extract common factors from the questionnaire items for employee and job satisfaction, and reliability was confirmed by Cronbach’s alpha coefficient [17]. IBM SPSS Statistics, version 28.0 (IBM Corp., Armonk, NY, USA), was used for statistical analysis. Furthermore, statistical hypothesis testing was two-tailed, with a significance level of < 5%.

### Ethical approval and consent to participate

This study was conducted through a completely anonymous survey. Data obtained were statistically processed so that individuals cannot be identified, and responses were not used for any purpose other than this survey. Third-party access to the survey was not and will not be given, and responses would not affect the individual’s institutional affiliation. Moreover, informed consent was obtained from all respondents who participated in the study as indicated in the survey form. Those terms and conditions were included in the survey form in accordance with the instructions of the Ethics Committee of TCROSS Co., Ltd. The study was conducted following the approval of the Ethics Committee TCROSS Co., Ltd (February 23, 2023, Approval No. 2023002). All procedures were followed as per the relevant guidelines (e.g., Declaration of Helsinki) and authors had access to information that could identify individual participants during or after data collection.

## Results

### Respondent attributes

From April 22, 2023, to May 21, 2023, 156 cardiologists completed the survey. The attributes of the respondents are shown in Table 1. No significant difference in age groups was found between medical doctors who reported “no affiliation with a medical office” (non-affiliated group, *n* = 39) and “affiliation with a medical office” (affiliated group, *n* = 117). In addition, no significant differences were found between the two groups by position in the hospital. The proportion of those in the clinic/hospital type working at a city/general hospital was higher in the non-affiliated group than in the affiliated group, whereas the proportion of those working at a university hospital was significantly higher in the affiliated group. Compared with the non-affiliated group, the affiliated group was significantly more likely to work at a hospital with > 500 beds.

**Table 1:** Subject attributes

	Total (*n* = 156)	Non-affiliated group (*n* = 39)	Affiliated group (*n* = 117)	*P*-value
**Age groups**				
Less than 30	7 (4.5%)	1 (2.6%)	6 (5.1%)	0.250
30–39	50 (32.1%)	8 (20.5%)	42 (35.9%)	
40–49	54 (34.6%)	18 (46.2%)	36 (30.8%)	
50–59	34 (21.8%)	8 (20.5%)	26 (22.2%)	
More than 60	11 (7.1%)	4 (10.3%)	7 (6.0%)	
**Job titles**				
Others	8 (5.1%)	2 (5.1%)	6 (5.1%)	0.500
Medical staff	48 (30.8%)	10 (25.6%)	38 (32.5%)	
Assistant director	44 (28.2%)	12 (30.8%)	32 (27.4%)	
Director	39 (25.0%)	8 (20.5%)	31 (26.5%)	
Vice president/president	17 (10.9%)	7 (17.9%)	10 (8.5%)	
**Clinic/hospital types**				
Clinic without bed	8 (5.1%)	3 (7.7%)	5 (4.3%)	0.014
Clinic/hospital with beds	4 (2.6%)	1 (2.6%)	3 (2.6%)	
City/general hospital	112 (71.8%)	34 (87.2%)	78 (66.7%)	
University hospital	32 (20.5%)	1 (2.6%)	31 (26.5%)	
**Beds**				
None	8 (5.1%)	3 (7.7%)	5 (4.3%)	< 0.001
1–19 beds	1 (0.6%)	0 (0)	1 (0.9%)	
20–99 beds	7 (4.5%)	6 (15.4%)	1 (0.9%)	
100–199 beds	15 (9.6%)	2 (5.1%)	13 (11.1%)	
200–299 beds	16 (10.3%)	9 (23.1%)	7 (6.0%)	
300–499 beds	54 (34.6%)	14 (35.9%)	40 (34.2%)	
500–699 beds	32 (20.5%)	4 (10.3%)	28 (23.9%)	
More than 700 beds	23 (14.7%)	1 (2.6%)	22 (18.8%)	
**Length of service**				
Less than 1 year	26 (16.7%)	9 (23.1%)	17 (14.5%)	0.119
1–3 years	35 (22.4%)	4 (10.3%)	31 (26.5%)	
4–6 years	30 (19.2%)	8 (20.5%)	22 (18.8%)	
7–9 years	20 (12.8%)	8 (20.5%)	12 (10.3%)	
More than 10 years	45 (28.8%)	10 (25.6%)	35 (29.9%)	

The percentage represents the overall proportion

### Actual condition of the work environment

The survey items shown in Table 2 were answered on a 5-point Likert scale. The non-affiliated group was more likely than the affiliated group to work at a hospital that emphasizes philosophy, and the number of medical doctors who responded to the organizational commitment item, particularly “I see hospital problems as my own problems,” was significantly higher in the non-affiliated group.

**Table 2:**
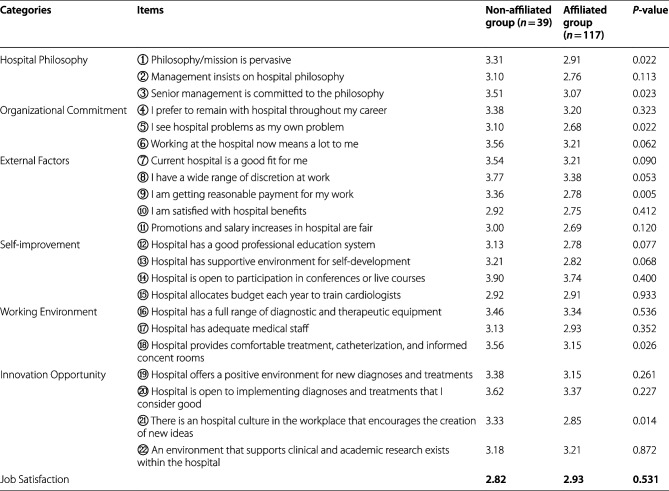
External and internal factors in the work environment

Values are score scale from 1 to 5

Remuneration, which plays an important role in external factors, was higher in the non-affiliated group than in the affiliated group. The work environment such as the catheterization room, which is considered crucial for cardiologists, was more satisfactory for the non-affiliated group than the affiliated group. Furthermore, cardiologists working in “workplaces that encourage creative work and the generation of new ideas” scored significantly higher in the non-affiliated group than the affiliated group. Despite these facts, no significant difference in job satisfaction was determined between the non-affiliated and affiliated groups (2.82 vs. 2.93, *p* = 0.531).

### Type of hospital management

Factor analysis was conducted to generate common factors from the items in Table 2 to detect the common management style behind them, and four factors were extracted. Items with factor loadings greater than 0.4 were combined to form each factor. Each item that made up a factor is summarized in Table 3. Factor 1 – 4 were defined as “environment-oriented hospitals,” “loyalty building-oriented hospitals,” “treatment-oriented hospitals,” and “philosophy-oriented hospitals” and accounted for 20.5%, 13.5%, 11.6%, and 10.3% of the total contribution, respectively (55.9% cumulative contribution). For example, “environment-oriented hospital” is considered a hospital comprising ten elements (12, 13, and 15–22) as shown in Table 3. A reliability test analysis was conducted with Cronbach’s alpha of α = 0.899, 0.869, 0.803, and 0.803 for factors 1–4, respectively, as an indication that the model is reliable (Table 3). The results of comparing the means of the factor scores of the respondents generated from the factor analysis between the groups (Table 4) showed that the means of all four management types were higher in the non-affiliated group than in the affiliated group. In particular, the means were significantly higher in the non-affiliated group in the philosophy-oriented hospital (*p* = 0.028) and the treatment-oriented hospital (*p* = 0.084).

**Table 3:**
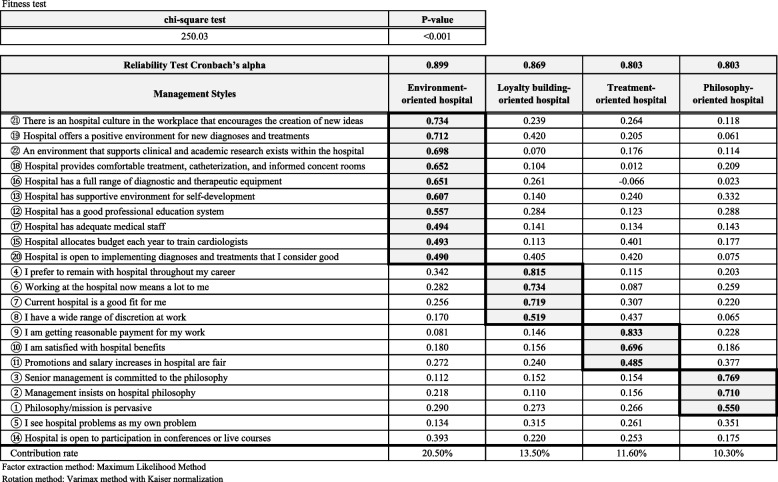
Common internal and external factors in the work environment (factor analysis)

**Table 4:** Comparison of the non-affiliated group and the affiliated group with the hospital management style

	Non-affiliated group (*n* = 39)	Affiliated group (*n* = 117)	*P*-value
Environment-oriented hospital	0.1026	-0.0342	0.424
Loylalty building-oriented hospital	0.1049	-0.0350	0.412
Treatment-oriented hospital	0.2210	-0.0737	0.084
Philosophy-oriented hospital	0.2621	-0.0874	0.028

Values use the mean of the factor scores

## Discussion

This study shows specific effects of the medical office system of Japan. First, compared with those who belonged to a university medical office, medical doctors who did not belong to a university medical office tended to work in hospitals that emphasized the importance of the hospital’s philosophy, had higher levels of commitment to the organization, and were in an environment that encouraged innovation. However, no evidence shows that these factors affected job satisfaction as shown in Table 2. Four types of hospital management styles were identified: namely, environment-oriented, loyalty-building, treatment-oriented, and philosophy-oriented hospitals, which were generated from 22 questions related to management philosophy, organizational commitment, external factors, self-improvement, work environment, and opportunities for innovation (Table 3). The non-affiliated group was more likely to work at a treatment-oriented hospital than the affiliated group (*p* = 0.084), and the difference was significant for the philosophy-oriented hospital (*p* = 0.028) as presented in Table 4.

### Insights from the medical office system; internal and external factors

In this study, some internal and external factors were significantly higher in the work environment of the non-affiliated group than the affiliated group (Table 2). When a medical doctor belongs to a medical office system, his/her place of employment is determined under the human resource management of the medical office system, which means that the medical doctor can be assigned to a non-preferred hospital while he/she is still young. Legally, the “free will of the individual (the young doctor being sent)” will be respected assumingly; however, owing to the long-standing traditions and structure of the medical office system and its affiliated hospitals, a medical doctor cannot easily refuse as long as he/she remains a member of the medical office [18]. The human resources of university medical offices play crucial role in securing medical doctors for affiliated hospitals. According to the results of a survey by the Japan Medical Association on the number of medical doctors needed at hospitals, more than 75% of hospitals in Japan continue relying on the university medical office system to secure medical doctors [19].

Conversely, members of the non-affiliated group can freely choose where they work, considering compensation, work environment, work–life balance, holidays, etc., when selecting a hospital that fits their requirements. Hospitals not affiliated with university medical offices will try to secure medical doctors by offering favorable conditions. According to the Central Social Insurance Medical Council’s “Report on the 23rd Annual Survey of Medical Economy” that was conducted in 2021, the annual income for men in their 30 s is 13 million yen at university hospitals, compared with 15 and 18 million yen at other hospitals and clinics, respectively, an increase of 15%–38% [20]. There is a difference between the salaries of medical doctors who belong and do not belong to a medical office, since they can freely choose a hospital. Small- and medium-sized private hospitals, that are not affiliated with medical offices, can pursue efficiency in their management, which allows them to enhance their medical facilities, making them a suitable environment for medical doctors whose mission is to save patients. For medical doctors who have chosen to leave the brand of their university’s medical office, they are interested in working in a comfortable environment with satisfactory compensation at a hospital that matches their skill level, rather than the name, or size of their institution.

### Management style of small- and medium-sized hospitals: philosophy-oriented and treatment-oriented hospitals

Of the four factors that were extracted from the factor analysis, philosophy-oriented hospitals were the only ones that reached significance between the non-affiliated and affiliated groups.

The hospital organization is a group of professionals with different qualifications, such as medical doctors, nurses, pharmacists, and technicians, and their values are “influenced by external authorities” [21]. A for-profit organization primarily pursues profit, whereas a non-profit hospital organization must share common values with its employees, rather than profit, to unite a group of people who are qualified in different fields of expertise. Unlike national, public, and university hospitals, managers of private hospitals cannot afford to leave their businesses running at a loss. However, the mission of medical doctors as professionals is to provide optimal medical care to each patient using their skills and techniques, and it is not their true desire to provide daily medical care with the interests of the hospital or organization in mind. The relationship between the two is influenced by the “mismatch of objectives” between the principal (hospital management) and the agent (medical doctor), as evidenced by agency theory, and “information asymmetry” that intervenes between the parties is a major factor [22].

Although the president of the hospital is a medical doctor, he/she lacks the level of knowledge regarding medical equipment and drugs used by frontline medical doctors due to differences in their fields of expertise. Undeniably, the “mismatch of objectives” and “asymmetry of information” between hospital management and frontline medical doctors is increasing, and medical doctors may practice medicine to the detriment of hospital management.

The importance of management philosophy was to overcome this challenge, particularly in small-, and medium-sized private hospitals, where philosophy is positioned as a means to control the organization and efforts are made to move employees in the same direction as the management team [21]. The expectation is that a philosophy will “unify the consciousness of employees,” “increase their motivation to work,” and increase their “commitment to the organization.” Thus, the study reveals the reality of uniting the profession around a philosophy-oriented management for small- and medium-sized hospitals that employ medical doctors away from the medical office system.

### Study limitations

The study examined the effect of the medical office system on job satisfaction, loyalty, engagement, and organizational commitment among cardiologists, and the results do not reflect medical doctors as a whole. The survey was conducted among cardiologists registered with a highly specialized news site. Moreover, approximately 70% of the respondents were in their 30 s and 40 s, which is a limitation of the survey’s target audience of cardiologists on the front lines of acute care medicine. Finally, the medical office system is unique to Japan and differs from international systems and is, therefore, not generic to other countries.

## Conclusions

Although the medical office system did not affect job satisfaction, compared with medical doctors dispatched by medical offices, those who voluntarily selected their employers tended to work in hospitals that emphasized philosophy-oriented management. Furthermore, they received high loyalty and moderate compensation while practicing in an environment suitable for their specialty. The results also suggest that medical doctors who are not affiliated with a medical office have high organizational commitment and are more likely to agree with the management philosophies set forth by hospital executives. These results suggest that the medical office system makes it difficult for medical doctors to have high workplace loyalty, engagement, and commitment to the hospital to which they are dispatched.

## Data Availability

The data that support the findings of this study is available on request from the corresponding author.
